# Bioinformatics and HIV Latency

**DOI:** 10.1007/s11904-014-0240-x

**Published:** 2015-01-14

**Authors:** Angela Ciuffi, Pejman Mohammadi, Monica Golumbeanu, Julia di Iulio, Amalio Telenti

**Affiliations:** 1 Institute of Microbiology, University Hospital of Lausanne (CHUV), University of Lausanne, Bugnon 48, 1011 Lausanne, Switzerland; 2 Department of Biosystems Science and Engineering, ETH Zurich, Basel, Switzerland; 3 Swiss Institute of Bioinformatics, Basel, Switzerland; 4 Department of Genetics, Harvard Medical School, Boston, MA USA; 5 Genetic Medicine, J. Craig Venter Institute, La Jolla, CA 92037 USA

**Keywords:** HIV, Latency, Bioinformatics, Integration, Transcription, Transcriptome, Proteome

## Abstract

Despite effective treatment, HIV is not completely eliminated from the infected organism because of the existence of viral reservoirs. A major reservoir consists of infected resting CD4+ T cells, mostly of memory type, that persist over time due to the stable proviral insertion and a long cellular lifespan. Resting cells do not produce viral particles and are protected from viral-induced cytotoxicity or immune killing. However, these latently infected cells can be reactivated by stochastic events or by external stimuli. The present review focuses on novel genome-wide technologies applied to the study of integration, transcriptome, and proteome characteristics and their recent contribution to the understanding of HIV latency.

## Introduction

The study of biological processes, including viral infections, benefits from novel technologies for exploration and discovery. In the past decade, new developments have focused on genome-wide analyses, including high-throughput sequencing technologies for genome and transcriptome analyses, as well as mass spectrometry-based technologies for proteomic studies. These technologies aim at providing the most comprehensive snapshot picture of a specific cellular content, thereby generating large amounts of data. In turn, large-scale datasets require the development of tools and methods to handle and analyze these data adequately. This review focuses on experimental and bioinformatic methodologies applied to the topic of HIV latency.

## HIV Latency

HIV infection is considered today a chronic disease, thanks to the efficacy of antiretroviral therapy (ART) [1, 2•, 3–7]. ART aims at blocking multiple viral replication steps and enzymatic activities, i.e., entry, reverse transcriptase, integrase, and protease, thereby limiting virus escape through selection of variants. ART results in undetectable viremia in the plasma of HIV+ individuals but fails to achieve complete virus eradication, hence requiring life-long treatment [1, 2•, 3–7]. HIV persistence is explained by the existence of reservoirs that are established early upon HIV exposure [8••, 9–11]. Two types of reservoirs have been described that are not mutually exclusive and that are likely to co-exist in some individuals. The first reservoir consists in anatomical sanctuaries, such as the central nervous system, gut, or lungs, where ART drug concentration may be suboptimal due to incomplete penetration [12–14]. Infection may persist in these sites due to either partial inhibition of viral replication or to cell-to-cell spread of the virus [12, 13, 15]. The second reservoir relies on the persistence of HIV in long-lived cells in a latent and thus “hidden” state. This relies on HIV’s ability to stably insert its genome in the host cell chromosome, thereby ensuring its life-long association with the infected host cell. This latent cell reservoir consists mostly of resting memory CD4+ T cells, but other cells have been shown to contribute as well, including naïve CD4+ T cells, macrophages, dendritic cells, and hematopoietic progenitor cells [16–22]. Latently infected CD4+ T cells can be induced, i.e., reactivated, and are thus able to produce particles through stochastic transcription or through immune activation, thereby leading to intermittent detectable viremia or viremia rebound after ART cessation [2•, 3, 23••].

The reservoir of latently infected CD4+ T cells is currently considered to be the major obstacle to HIV cure. Global scientific efforts have focused on infected CD4+ T cells in order to understand the nature of latency, i.e., nature of post-integration blocks hampering viral particle production, as well as designing new strategies to stimulate cells to exit latency. For this purpose, multiple models have been developed to characterize various mechanisms that can contribute to HIV latency [24, 25]. Models have also been useful to test reactivation compounds inducing viral particle production from the latently infected CD4+ T cells [26••, 27–29]. The present review focuses on recent genome-wide analyses performed on the models of HIV latency (Table 1).

**Table 1:** Genome-wide analyses on HIV latency models

HIV latency models	Analysis type	Experimental approach	Computational tools/approaches	Reference
T cell lines	Integration	DNA digestion (AvrII/XbaI/SpeI or MseI) Linker ligation First PCR (LTR-linker) Nested PCR TOPO cloning and transformation Sanger sequencing	Trimming: custom Perl scripts Genome alignment: BLAT Integration site inclusion criteria: starting at junction with HIV LTR, mapping to the human genome reference sequence with >98 % identity Annotation databases: RefSeq (Acembly, GenScan, Unigene)	Lewinski et al. [51] (Sherrill-Mix et al. [42••])
	Proteome	Two-dimensional gel electrophoresis matrix-assisted laser desorption/ionization-time-of-flight (MALDI-TOF)	Protein identification: ProFound, Prospector, and Mascot software	Berro et al. [95]
	Proteome	Solid-phase extraction of glycopeptides (SPEG) High-performance liquid chromatography-tandem mass spectrometry (HPLC-MS/MS)	Raw data quality evaluation: Trans-Proteomic Pipeline (TPP) Peptide evaluation: INTERACT and Peptide Prophet Label-free quantitation with MaxQuant identification of differentially expressed glycoproteins using a twofold cutoff ratio and a Student’s *t* test with *p* value ≤0.05	Yang et al. [96]
Bcl-2-modified CD4+ T cell model	Integration	DNA digestion (PstI) Dilution and circularization by ligation Inverse PCR amplification Sequencing	Sequence alignment using the UCSC Bioinformatics Human Genome database Integration site inclusion criteria: HIV LTR sequence	Shan et al. [54] Sherrill-Mix et al. [42••]
CD4+ T cell co-culture with H80 feeder model	Transcriptome	Total RNA extraction Spike-in addition (ERCC Exfold) mRNA-Seq library preparation (TruSeq) mRNA sequencing (Illumina HiSeq 2000)	Trimming: Cutadapt (Illumina adapter removal) Quality filtering: prinseq (low quality and PHRED score reads filtering) Genome and transcriptome alignment: Tophat Differential expression analysis: DESeq Enrichment tests: Fisher’s exact test Online visualization tool: LITCHi^1^ (GuavaH^2^)	Mohammadi et al. [32••]
Direct infection of resting CD4+ T cells	Integration	DNA digestion (Tsp509I or MseI) Linker ligation First PCR (LTR-linker) Nested PCR with barcoded primers containing sequencing adapters 454 sequencing	Integration analysis done by web-based tool InSiPiD^3^ Trimming Genome alignment: BLAT, hg18 Integration site inclusion criteria: LTR sequence, mapping to the human genome reference sequence within the first 3 base pairs (bp) and a unique alignment with >98 % identity Annotation database: RefSeq Statistical analysis: one-tailed Student’s *t* test, one-way ANOVA, logistic regression	Pace et al. [52] Sherrill-Mix et al. [42••]
	Integration	DNA digestion (MseI) Linker ligation First PCR (LTR-linker) Nested PCR with barcoded primers containing sequencing adapters Ion Torrent sequencing (Ion PGM 200 sequencing, Ion 316 chip)	Integration analysis done by web-based tool InSiPiD^3^ Trimming: hiReadsProcessor R package Genome alignment: BLAT, hg19 Integration site inclusion criteria: LTR and primer sequences, mapping to the human genome reference sequence within the first 3 bp and with 98 % identity, quality score >15 for sequences shorter than 24 bases Annotation databases: UCSC, Encode, RNA-Seq. ChIP-Seq Statistical analysis: *R*	Sherrill-Mix et al. [42••]
	Transcriptome	RNA extraction RNA amplification Hybridization to Illumina Human-Ref8 (v3) BeadChip (microarray) Chip scanning with Illumina BeadStation 500GX scanner using Illumina BeadStudio (version 3) software	Raw data processing and filtering using Bioconductor (R), Benjamini and Hochberg *p* value correction with a 0.05 false discovery rate Analysis: Limma package (R) Pathway enrichment analysis: Ingenuity Pathway Analysis (IPA) software	Evans et al. [78]
Patient-derived ex vivo model	Integration	DNA shearing (Covaris Adaptive Focused Acoustics) into 300–500-bp fragments End repair (Epicentre End-it DNA End Repair) dA addition (NEB dA-tailing kit) Linker ligation First PCR (LTR-linker) Nested PCR with N6-barcoded primers containing sequencing adapters Illumina sequencing: 2 × 150-bp paired-end (MiSeq) or 2 × 105-bp paired-end (HiSeq)	Trimming: custom Perl scripts Genome alignment: BLAT, hg19 Integration site inclusion criteria for read 1: LTR primer sequence, 5 last bp of the LTR sequence followed with >20-bp DNA sequence with an average quality score >20, mapping to the human genome reference sequence within the first 3 bp and with >95 % identity Integration site inclusion criteria for read 2: alignment to the human genome reference sequence on the opposite strand compared to read 1 and within 1 kb Integration sites are considered different if the breaking point differs from >3 bp Gene ontology analysis: GREAT Statistical analysis: Fisher’s exact test	Maldarelli et al. [44•]
	Integration	Linear amplification using forward primer in HIV 3′ end (env or nef region) Second-strand synthesis using a random decamer primer tailed with HIV U5 sequence End repair by trimming (NEB exonuclease I first followed by MyTaq fill-in) Denaturation, annealing, and extension to generate a panhandle structure (LTR sequence double-stranded and loop single-stranded DNA as host DNA) PCR amplification with one specific LTR primer (R region) Nested PCR with inner LTR primer (R or U5 region) Sequencing using downstream U5 primer	Trimming: Sequencher Genome alignment: Bowtie2 (and BLAT), hg19 Annotation databases: RefSeq genes, UCSC known genes Gene ontology analysis: TopGO Statistical analysis: Fisher’s exact test, Holm-Bonferroni correction, Cochran-Armitage test	Wagner et al. [45•]

^1^ *LITCHi* latent infection of T cells by HIV (http://litchi.labtelenti.org/)

^2^ *GuavaH* genomic utility for association and viral analyses in HIV (http://www.guavah.org/) [100]

^3^ *InSiPiD* integration site pipeline and database (http://www.bushmanlab.org/tools)

## The Inducible Reservoir

Available methods overestimate or underestimate the size of the latent viral reservoir. This is mainly due to (i) the use of blood samples as the source of the latent reservoir, (ii) the major presence of cells carrying defective viral genomes which obscures the proportion of latently infected cells, and (iii) the incomplete success of current methods used to reactivate latently infected cells in vitro. Indeed, it has been estimated that out of one million blood-purified resting CD4+ T cells from ART-treated individuals, on average, ∼1000 cells (ranging approximately between 100 and 2000 cells) were infected by HIV and thus contained proviral DNA [30••]. However, only a small proportion of these HIV+ cells (11.7 %) carry replication-competent viral genome sequences and are thus inducible [31••]. Using phytohemagglutinin, interleukin-2, and irradiated peripheral blood mononuclear cells to stimulate CD4+ T cells in a viral outgrowth assay, only 1 % of HIV+ cells were successfully induced, thereby illustrating that current reactivation protocols and methods stimulate only a fraction of the total inducible reservoir [30••, 31••]. Other methods of stimulation using anti-CD3/CD28 and interleukin-7 for 7 days were able to induce particle production from 1.5 % of HIV+ cells (ranging from 0.6 to 2.4 % in the 13 patients tested) [23••]. Finally, reactivation of latently infected cells may occur stochastically and spontaneously at a frequency of 0.041 % (0.03–0.15 %) [23••].

These studies indicate that the latency is complex as stimulation does not lead to 100 % reactivation of latently infected cells [26••, 31••]. Cillo et al. also show that, under their stimulation conditions, 7.5 % of infected cells were expressing cell-associated RNA, an almost 40× increase in HIV transcription compared to unstimulated resting CD4+ T cells. The gap between the successful viral particle production and viral transcription suggests that transcriptional latency cannot recapitulate all aspects of the viral reservoir and that other post-integration blocks exist, consistent with multiple recent studies using latency-reactivating agents and multiple models of HIV latency [23••, 26••, 32••].

## Integration Site Location and HIV Latency

Reverse transcription of the viral RNA genome and its integration in the host cell chromosome are two hallmarks of retroviruses. In the past decade, thanks to the availability of the human genome sequence, many efforts have been focused on the understanding of the preferential site of HIV integration, as well as its consequences on the host cell, mostly regarding insertional mutagenesis [33–35]. HIV has been shown to integrate preferentially into active transcription units, with no preference for exons or introns, neither for orientation [34, 36, 37]. The site of integration may have consequences for both HIV transcription and host gene transcription because of chromatin arrangement and RNA interference [35, 38]. Therefore, the site of integration can affect the balance between viral transcriptional success and latency, as well as establish a balance between cell death and clonal expansion.

Analysis of integration site distribution commonly uses a three-step experimental approach: (i) DNA fragmentation (sonication, restriction enzymes), (ii) linker ligation, and (iii) PCR amplifications using primer annealing in the long terminal repeat (LTR) and primer annealing in the ligated linker [39–41]. The amplicons are sequenced using next-generation sequencing technologies, generating millions of short reads (ranging usually from ∼20 bp to a few hundreds of bp). Virus sequences are removed and trimmed sequences are aligned to the human genome to locate the integration site. This method allows the efficient capture of the preferential pattern of integration site distribution according to the human reference genome annotations. However, it fails to quantify the frequency of integration sites occurring at the exact same location due to the bias caused by the PCR amplification step [42••]. This limitation was recently addressed using random shearing of the DNA or through the usage of random decamer primers tailed with a U5 sequence that allowed discriminating between two identical integration site locations originating from an amplicon sequenced twice or from two individual integration events occurring at the same chromosomal position [43, 44•, 45•].

Maldarelli et al. and Wagner et al. observed that the same integration site was identified multiple times in latently infected cells, suggesting that a single infected cell was clonally expanded over time [44•, 45•]. The location of the proviral insertion in these clonally expanded infected cells was enriched in genes involved in cell division cycle or cancer, supporting the notion that the site of viral integration may promote homeostatic proliferation. It is important to note that, despite this observation, cancer due to HIV insertional mutagenesis is not a major issue in HIV-infected individuals [36, 46]. Alternative hypotheses explaining the observed cell clonal expansion include (i) the infection of stem cells [20, 21, 47, 48] and (ii) the possible survival advantage of cells carrying defective viral genomes [31••, 46]. It would thus be interesting to know if the expanded cell clones are part of the inducible or defective viral reservoir. This would guide viral eradication strategies to account for those cells and explore ways to specifically inhibit their proliferation.

The link between the integration site and HIV latency is considered to be at the transcriptional level, resulting in silencing via epigenetic regulation of the chromatin environment or transcriptional interference [25, 49, 50]. Spatial features of the integration site associated with transcriptional latency have been shown to favor (i) heterochromatin (centromeric alphoid repeats) and intergenic regions [51, 52], (ii) flanking cellular genes that are highly expressed [51, 53], and (iii) the same orientation as cellular genes [54]. In contrast, Dahabieh et al. suggested that NF-kB activity and cell activation state correlate with viral gene expression efficiency rather than genomic integration features [55].

The role of integration site location on HIV transcriptional activity has been recently assessed in five primary cell models of HIV latency, identifying features specific for each model [42••]. Viral silencing was stronger if the host gene expression was high in the Jurkat latent model and in active CD4+ T cells, while the contrary was observed in a central memory CD4+ T cell model. The same orientation integration bias in the Bcl-2 latency model was confirmed but was not identified in the other latency models [54]. Integration in intergenic regions was favored in active or resting CD4+ cell models only. In contrast, integration in alphoid repeats was preferentially identified in the Jurkat latency model, confirming a previous study, and in resting and central memory cell models [51]. Finally, only the H4K12ac acetylation epigenetic mark correlated with latency in active, resting, and central memory CD4+ cell models but not in the Bcl-2 and Jurkat cell line models. This study highlighted characteristics specific for each model but failed to identify common features of integration site selection specific of HIV latency across all latency models, leaving open the debate about the contribution of integration site location to transcriptional silencing [42••]. Single-cell analysis may facilitate understanding of the link existing between integration site location and viral transcription.

## Transcriptome and HIV Latency

Transcriptome analyses capture a snapshot of RNA molecules within the cell at a specific time. The mRNA molecules will be translated shaping the proteome content of the cell and therefore its functionality. Although the transcriptome only partially correlates with the proteome, it still provides a valuable approximation of the cell condition at a specific time [56–58]. The amount of specific mRNA species results from a balance between the production rate and decay rate. The former is determined by an interplay of regulatory processes acting at transcription initiation, elongation, and termination steps, and the latter is influenced by proteins binding to the 3′ untranslated region (3′ UTR) and the activity of regulatory RNAs such as microRNAs and noncoding RNAs [59–68]. External stimuli will affect this balance, tipping it towards one direction or another [69, 70]. An additional layer of complexity resides in alternative splicing, for both HIV and host cells [59, 71–73].

Transcriptional silencing of HIV has been considered as a major cause of latency [25, 49, 50]. The majority of studies investigating transcriptional silencing have focused on mechanisms acting on the viral LTR promoter, highlighting the role of the viral Tat protein, cellular proteins, histone post-translational modifications, and chromatin remodeling [25, 49, 50, 74–77]. However, only a few studies have focused on the transcriptome analysis of latently infected cells. Evans et al. developed a new primary model of HIV latency, which consists in co-culturing resting CD4+ T cells with myeloid dendritic cells (mDC) for 24 h, followed by CCR5-tropic HIV-GFP infection [78]. Latently infected cells were defined as nonproliferating CD4+ T cells not expressing viral-encoded GFP and were thus sorted by fluorescence-activated cell sorting (FACS) as SNARF-1^high^ and GFP^−^ cells after 5 days post-infection. This model provided evidence that CD4+ T cell direct contact with mDC, but not plasmacytoid DC (pDC), facilitates establishment of HIV latency. The study used microarrays to compare latently infected CD4+ T cells versus mock-infected CD4+ T cells co-cultured with mDC and identified only a limited number of genes, mostly interferon I-regulated genes (IFIT1, IFI27, and OAS1), as differentially expressed. These genes may represent latency biomarkers but may also originate from noninfected exposed cells.

Gene arrays are being replaced by high-throughput sequencing technologies, as these do not require prior knowledge about gene sequences and provide a higher accuracy. Current methods differ in the library preparation and in the targeted RNA species. For example, total RNA sequencing (including small RNAs) allows capturing a complete picture of RNA within a cell, coding or noncoding [79], but requires a high coverage and does not discriminate between nuclear immature transcripts and cytoplasmic mature transcripts. Messenger RNA-Seq (mRNA-Seq) is based on poly(A) capture, thereby requiring less sequencing depth, and will reflect more faithfully the coding transcriptome, although it will not account for coding transcripts that are not poly-adenylated [80, 81]. Recent improvements in library preparation protocols include determination of RNA strand specificity as well as of splicing variants of the coding transcripts. The analysis of these sequences requires computational tools to align the transcripts to the genome and identify transcript isoforms.

The analysis by Evans et al. highlighted the importance of analyzing purified populations of latently infected resting CD4+ T cells in order to identify specific latency biomarkers [78]. Because of the difficulty to isolate and obtain an uncontaminated population of latently infected cells, only a limited number of studies investigated the cellular transcriptome of HIV latency in primary models. Mohammadi et al. used a primary CD4+ T cell model infected with an attenuated viral construct. Successfully infected cells were isolated and purified by FACS and allowed to revert to a resting, hence latent state, by co-culture with H80 feeder cells [32••]. They analyzed the transcriptome of the infected cells over time by mRNA-Seq, thereby following establishment of latency. Cellular activation or resting state was shown to be a major driver of transcriptional differences between cells. Except for the persistent detection of viral transcripts, only a very limited number of host genes were found to be differentially expressed between noninfected and latently infected cells, suggesting that HIV had a very minor impact on the transcriptome of the resting cell. Latently infected cells were also exposed for 8 and 24 h to diverse stimuli to reactivate latency, including SAHA (vorinostat), disulfiram, and IL-7. Although some of these molecules were impacting viral transcription, they failed to stimulate viral protein expression, underscoring the possibility that post-transcriptional blocks would also contribute to the latent phenotype. This study indicated that HIV latency reflects transcriptional and post-transcriptional blocks, including nuclear export and translation [26••, 32••, 82]. The multiplicity of mechanisms leading to HIV latency was also illustrated recently by the comparison of several latency-reactivating agents and their different efficiencies across latency models [26••].

## Proteome and HIV Latency

The genetic flow of information in the cell attains a key principal level with the translation of messenger RNA into functional protein. Each protein is modulated through tissue-specific dynamic processes such as alternative splicing, post-translational modifications, interactions with other molecules, and formation of proteomic complexes, which delineate the multidimensional complex nature of the proteome. A recently published blueprint of the human proteome reported approximately 3 × 10^6^ unique peptides covering 84 % of the annotated coding genome [83]. Analyzing the proteins of a biological system gives the opportunity to understand and reveal the intricacies of many cellular functional pathways, inaccessible through genomic or transcriptomic studies.

During HIV infection, as in the case of any viral infection, the proteome of the infected host cell constitutes a key layer where a plethora of responses and interactions with the viral components take place [84, 85]. HIV orchestrates various cellular processes by interacting and manipulating the functional blocks on the host cell in order to promote viral replication and assure its survival [86]. On the host side, various studies have identified changes in post-translational modifications, localization, and amount of proteins upon infection by HIV [87, 88]. In addition to taking advantage of the host cellular proteome, HIV brings along an arsenal of 15 viral proteins that interfere with the host cell, establish interactions, and carry diverse post-translational modifications in order to assure key steps of the virus life cycle.

Recent technological advances and the advent of mass spectrometry (MS)-based proteomics have made a strong positive impact on virology and have helped uncover and characterize many proteomic responses triggered by viral infection [89, 90]. A standard MS experiment consists of a succession of steps: sample protein fractionation into smaller peptides, separation, ion detection, and relative or absolute protein quantitation. The general pipeline can be adapted to address more specific questions such as studying alterations in the phosphoproteome [91]. Online databases and search algorithms are used to map the obtained peptides to putative proteins. The resulting proteomic MS data has additional complexity since, often, more than one candidate protein is associated to the measurements from a set of peptides. A necessary step in MS data pre-processing is to filter out proteins with few, uncertain peptide measurements. MS experiments allow obtaining relative or absolute protein measurements between two different conditions (e.g., HIV-infected and mock-infected cells) which need to be normalized.

Jager et al. used affinity purification coupled with MS (AP-MS) to construct a map of HIV-host interactions at the proteome level. The authors devised a scoring system coined MiST which incorporates the reproducibility, abundance, and specificity of a protein to filter out nonspecific interactions. They reported on 497 high-confidence interactions, mainly enriched in transcription and regulation of viral protein post-translational modifications [92]. Another study employed isobaric tags for relative and absolute quantitation (iTRAQ) coupled with tandem MS to determine the host proteome response to HIV infection in a time-dependent manner [93]. Differentially expressed proteins were identified between HIV- and mock-infected samples at three different time points post-infection. Interestingly, the groups of differentially expressed proteins differed between early and late time points, capturing an early response enriched for cell proliferation, protein synthesis, DNA recombination, repair, and maintenance versus a late reaction linked to T cell activation [93]. Stable isotope labeling by amino acids in cell culture (SiLAC) profiling of phosphopeptides during HIV entry in the host cell has revealed responses to HIV infection at the level of the host phosphoproteome, notably suggesting that HIV affects the phosphorylation of serine-rich host proteins to ease its replication and subsequent release [94•].

A series of host transcription factors, such as NF-kB, NFAT, or Sp1, bind to the HIV promoter, thereby modulating viral transcriptional activity in infected CD4+ T cells. Their nuclear availability varies between active and resting, latently infected cells [25, 95, 96]. Furthermore, reversible post-translational modifications of histones, such as deacetylation, have been found to play an important role in the establishment and maintenance of latency by modeling the neighboring chromatin organization at the viral LTR promoter [25, 27, 49, 50]. At the membrane level, 17 proteins involved in cell survival pathways or in the regulation of cellular adherence and transfer were found to be disrupted in latently infected cells [97]. The same study also showed that targeted treatment with inhibitors of several of these proteins, precisely XIAP and BTK, influenced the viability of latently infected cells [97]. Understanding the molecular pathways contributing to HIV latency at the proteome level may help in designing novel treatments to eliminate the quiescent virus.

Little is known about HIV-host interactions and the host proteome response during latency and reactivation. To date, no study has compared the whole proteomes of latent and activated HIV-infected cells. Glycoproteomic tandem MS has been employed to profile the disruption in glycoprotein secretion from latently HIV-infected T cells and to identify aberrantly secreted proteins in plasma from infected patients. Six glycoproteins, l-selectin, neogenin, galectin-3-binding protein, CECR1, ICOS, and the phospholipid transfer protein, were found to be overly secreted by latently infected T cells and elevated in patient plasma [98]. Although the mechanisms through which these proteins contribute to HIV latency remain elusive, this study represents a starting point to explore how latency alters protein secretion of the host cell. Another recent study focused on characterizing histone post-translational modifications in SupT1 cell lines infected with HIV, UV-inactivated HIV, and mock-infected in order to find a link to the transcriptional behavior of the cell. Using nano-LC-MS/MS, a set of histone modifications was detected in HIV- versus mock-infected cells, providing insight on how the virus modulates chromatin accessibility to assure its transcription during infection [99]. This study illustrates the epigenetic mechanisms used to manipulate chromatin to ensure transcriptional silencing during latency. Improvements of measurement investigation techniques and joint analyses of all post-translational modifications should help in assembling a complete view of the whole cellular proteome, thereby informing on the identity and the role of specific modified proteins in the process of latency.

## Conclusions

Unraveling the different aspects of latency will be a major step towards efficient HIV treatment and eradication. However, HIV latency and its reactivation have proven to be complex processes with many confounding factors. Genome-wide data provide a snapshot of the latently infected cell, at different molecular levels, i.e., integration site, transcriptome, and proteome. However, several aspects may influence the analysis: (i) The resting status of the cell limits the possibility of identifying unique markers of latency, (ii) the presence of a large number of infected cells carrying defective viral genome sequences obscures the features of the latent viral reservoir that can be reactivated with the goal of cure, and (iii) the analysis of high-throughput genome-wide data is particularly challenging due to the large amount of data as well as systematic technological and biological noise that may lead to high false-positive rates. Adequate bioinformatic tools are required to account for these effects in order to process and analyze the data. In-depth and dynamic genome-wide studies can provide essential contributions to the understanding of establishment of latency during resting condition, maintenance of latency, and the steps towards full reactivation.

## References

[1] Siliciano JD, Siliciano RF (2006). The latent reservoir for HIV-1 in resting CD4+ T cells: a barrier to cure. Curr Opin HIV AIDS.

[2.•] Durand CM, Blankson JN, Siliciano RF (2012). Developing strategies for HIV-1 eradication. Trends Immunol.

[3] Barouch DH, Deeks SG (2014). Immunologic strategies for HIV-1 remission and eradication. Science.

[4] Barton KM, Burch BD, Soriano-Sarabia N, Margolis DM (2013). Prospects for treatment of latent HIV. Clin Pharmacol Ther.

[5] Abou-El-Enein M, Bauer G, Reinke P, Renner M, Schneider CK (2014). A roadmap toward clinical translation of genetically-modified stem cells for treatment of HIV. Trends Mol Med.

[6] Kent SJ, Reece JC, Petravic J, Martyushev A, Kramski M, De Rose R (2013). The search for an HIV cure: tackling latent infection. Lancet Infect Dis.

[7] Coiras M, Lopez-Huertas MR, Perez-Olmeda M, Alcami J (2009). Understanding HIV-1 latency provides clues for the eradication of long-term reservoirs. Nat Rev Microbiol.

[8.••] Whitney JB, Hill AL, Sanisetty S, Penaloza-MacMaster P, Liu J, Shetty M, et al. Rapid seeding of the viral reservoir prior to SIV viraemia in rhesus monkeys. Nature. 2014;512:74–7. *This study shows that upon viral infection the viral reservoir is established very early (in less than three days) in an animal model. This study also shows that HIV is detectable in lymph nodes but not in blood of some animals, suggesting that lymph nodes may be an early and important reservoir*. 10.1038/nature13594 PMC4126858 25042999

[9] Zaikos TD, Collins KL (2014). Long-lived reservoirs of HIV-1. Trends Microbiol.

[10] Eisele E, Siliciano RF (2012). Redefining the viral reservoirs that prevent HIV-1 eradication. Immunity.

[11] Coleman CM, Wu L (2009). HIV interactions with monocytes and dendritic cells: viral latency and reservoirs. Retrovirology.

[12] Fletcher CV, Staskus K, Wietgrefe SW, Rothenberger M, Reilly C, Chipman JG (2014). Persistent HIV-1 replication is associated with lower antiretroviral drug concentrations in lymphatic tissues. Proc Natl Acad Sci U S A.

[13] Costiniuk CT, Jenabian MA (2014). Cell-to-cell transfer of HIV infection: implications for HIV viral persistence. J Gen Virol.

[14] Gray LR, Roche M, Flynn JK, Wesselingh SL, Gorry PR, Churchill MJ (2014). Is the central nervous system a reservoir of HIV-1?. Curr Opin HIV AIDS.

[15] Denton PW, Long JM, Wietgrefe SW, Sykes C, Spagnuolo RA, Snyder OD (2014). Targeted cytotoxic therapy kills persisting HIV infected cells during ART. PLoS Pathog.

[16] Soriano-Sarabia N, Bateson RE, Dahl NP, Crooks AM, Kuruc JD, Margolis DM, et al. The quantitation of replication-competent HIV-1 in populations of resting CD4+ T cells. J Virol. 2014; 88:14070-7. 10.1128/JVI.01900-14 PMC4249150 25253353

[17] Kumar A, Abbas W, Herbein G (2014). HIV-1 latency in monocytes/macrophages. Viruses.

[18] Bacchus C, Cheret A, Avettand-Fenoel V, Nembot G, Melard A, Blanc C (2013). A single HIV-1 cluster and a skewed immune homeostasis drive the early spread of HIV among resting CD4+ cell subsets within one month post-infection. PLoS ONE.

[19] Archin NM, Bateson R, Tripathy MK, Crooks AM, Yang KH, Dahl NP (2014). HIV-1 expression within resting CD4+ T cells after multiple doses of vorinostat. J Infect Dis.

[20] Nixon CC, Vatakis DN, Reichelderfer SN, Dixit D, Kim SG, Uittenbogaart CH (2013). HIV-1 infection of hematopoietic progenitor cells in vivo in humanized mice. Blood.

[21] McNamara LA, Onafuwa-Nuga A, Sebastian NT, Riddell J, Bixby D, Collins KL (2013). CD133+ hematopoietic progenitor cells harbor HIV genomes in a subset of optimally treated people with long-term viral suppression. J Infect Dis.

[22] Narasipura SD, Kim S, Al-Harthi L (2014). Epigenetic regulation of HIV-1 latency in astrocytes. J Virol.

[23.••] Cillo AR, Sobolewski MD, Bosch RJ, Fyne E, Piatak M, Coffin JM (2014). Quantification of HIV-1 latency reversal in resting CD4+ T cells from patients on suppressive antiretroviral therapy. Proc Natl Acad Sci U S A.

[24] Pace MJ, Agosto L, Graf EH, O’Doherty U (2011). HIV reservoirs and latency models. Virology.

[25] Hakre S, Chavez L, Shirakawa K, Verdin E (2012). HIV latency: experimental systems and molecular models. FEMS Microbiol Rev.

[26.••] Spina CA, Anderson J, Archin NM, Bosque A, Chan J, Famiglietti M (2013). An in-depth comparison of latent HIV-1 reactivation in multiple cell model systems and resting CD4+ T cells from aviremic patients. PLoS Pathog.

[27] Shirakawa K, Chavez L, Hakre S, Calvanese V, Verdin E (2013). Reactivation of latent HIV by histone deacetylase inhibitors. Trends Microbiol.

[28] Archin NM, Margolis DM (2014). Emerging strategies to deplete the HIV reservoir. Curr Opin Infect Dis.

[29] Xing S, Siliciano RF (2013). Targeting HIV latency: pharmacologic strategies toward eradication. Drug Discov Today.

[30.••] Eriksson S, Graf EH, Dahl V, Strain MC, Yukl SA, Lysenko ES (2013). Comparative analysis of measures of viral reservoirs in HIV-1 eradication studies. PLoS Pathog.

[31.••] Ho YC, Shan L, Hosmane NN, Wang J, Laskey SB, Rosenbloom DI (2013). Replication-competent noninduced proviruses in the latent reservoir increase barrier to HIV-1 cure. Cell.

[32.••] Mohammadi P, di Iulio J, Munoz M, Martinez R, Bartha I, Cavassini M (2014). Dynamics of HIV latency and reactivation in a primary CD4+ T cell model. PLoS Pathog.

[33] Papayannakos C, Daniel R (2013). Understanding lentiviral vector chromatin targeting: working to reduce insertional mutagenic potential for gene therapy. Gene Ther.

[34] Ciuffi A, Bushman F: Retroviral integration target site selection. In: HIV-1 integrase: mechanism of action and inhibitor design. edn 1. Edited by Neamati N: John Wiley & Sons, Inc.; 2011:51–65. [Binghe W (Series Editor): Wiley Series in Drug Discovery and Development

[35] Desfarges S, Ciuffi A: Viral integration and consequences on host gene expression. In: Viruses: essential agents of life. Edited by Witzany G: Springer Science + Business Media; 2012.

[36] Ciuffi A (2008). Mechanisms governing lentivirus integration site selection. Curr Gene Ther.

[37] Desfarges S, Ciuffi A (2010). Retroviral integration site selection. Viruses.

[38] Ciuffi A, Telenti A (2013). State of genomics and epigenomics research in the perspective of HIV cure. Curr Opin HIV AIDS.

[39] Ciuffi A, Barr SD (2011). Identification of HIV integration sites in infected host genomic DNA. Methods.

[40] Ciuffi A, Ronen K, Brady T, Malani N, Wang G, Berry CC (2009). Methods for integration site distribution analyses in animal cell genomes. Methods.

[41] Gabriel R, Eckenberg R, Paruzynski A, Bartholomae CC, Nowrouzi A, Arens A (2009). Comprehensive genomic access to vector integration in clinical gene therapy. Nat Med.

[42.••] Sherrill-Mix S, Lewinski MK, Famiglietti M, Bosque A, Malani N, Ocwieja KE (2013). HIV latency and integration site placement in five cell-based models. Retrovirology.

[43] Firouzi S, Lopez Y, Suzuki Y, Nakai K, Sugano S, Yamochi T (2014). Development and validation of a new high-throughput method to investigate the clonality of HTLV-1-infected cells based on provirus integration sites. Genome Med.

[44.•] Maldarelli F, Wu X, Su L, Simonetti FR, Shao W, Hill S (2014). HIV latency. Specific HIV integration sites are linked to clonal expansion and persistence of infected cells. Science.

[45.•] Wagner TA, McLaughlin S, Garg K, Cheung CY, Larsen BB, Styrchak S (2014). HIV latency. Proliferation of cells with HIV integrated into cancer genes contributes to persistent infection. Science.

[46] Margolis D, Bushman F (2014). HIV/AIDS. Persistence by proliferation?. Science.

[47] Buzon MJ, Sun H, Li C, Shaw A, Seiss K, Ouyang Z (2014). HIV-1 persistence in CD4+ T cells with stem cell-like properties. Nat Med.

[48] Powers JM, Trobridge GD. Identification of hematopoietic stem cell engraftment genes in gene therapy studies. J Stem Cell Res Ther. 2013;2013. 10.4172/2157-7633.S3-004 PMC3875223 24383045

[49] Van Lint C, Bouchat S, Marcello A (2013). HIV-1 transcription and latency: an update. Retrovirology.

[50] Mbonye U, Karn J (2014). Transcriptional control of HIV latency: cellular signaling pathways, epigenetics, happenstance and the hope for a cure. Virology.

[51] Lewinski MK, Bisgrove D, Shinn P, Chen H, Hoffmann C, Hannenhalli S (2005). Genome-wide analysis of chromosomal features repressing human immunodeficiency virus transcription. J Virol.

[52] Pace MJ, Graf EH, Agosto LM, Mexas AM, Male F, Brady T (2012). Directly infected resting CD4+ T cells can produce HIV Gag without spreading infection in a model of HIV latency. PLoS Pathog.

[53] Han Y, Lassen K, Monie D, Sedaghat AR, Shimoji S, Liu X (2004). Resting CD4+ T cells from human immunodeficiency virus type 1 (HIV-1)-infected individuals carry integrated HIV-1 genomes within actively transcribed host genes. J Virol.

[54] Shan L, Yang HC, Rabi SA, Bravo HC, Shroff NS, Irizarry RA (2011). Influence of host gene transcription level and orientation on HIV-1 latency in a primary-cell model. J Virol.

[55] Dahabieh MS, Ooms M, Brumme C, Taylor J, Harrigan PR, Simon V (2014). Direct non-productive HIV-1 infection in a T-cell line is driven by cellular activation state and NFkappaB. Retrovirology.

[56] Ghazalpour A, Bennett B, Petyuk VA, Orozco L, Hagopian R, Mungrue IN (2011). Comparative analysis of proteome and transcriptome variation in mouse. PLoS Genet.

[57] Low TY, van Heesch S, van den Toorn H, Giansanti P, Cristobal A, Toonen P (2013). Quantitative and qualitative proteome characteristics extracted from in-depth integrated genomics and proteomics analysis. Cell Rep.

[58] Fu X, Fu N, Guo S, Yan Z, Xu Y, Hu H (2009). Estimating accuracy of RNA-Seq and microarrays with proteomics. BMC Genomics.

[59] Bentley DL (2014). Coupling mRNA processing with transcription in time and space. Nat Rev Genet.

[60] Groen JN, Capraro D, Morris KV (2014). The emerging role of pseudogene expressed non-coding RNAs in cellular functions. Int J Biochem Cell Biol.

[61] Groen JN, Morris KV (2013). Chromatin, non-coding RNAs, and the expression of HIV. Viruses.

[62] Swaminathan S, Kelleher AD (2014). MicroRNA modulation of key targets associated with T cell exhaustion in HIV-1 infection. Curr Opin HIV AIDS.

[63] Chiang K, Rice AP (2012). MicroRNA-mediated restriction of HIV-1 in resting CD4+ T cells and monocytes. Viruses.

[64] Fowler L, Saksena NK (2013). Micro-RNA: new players in HIV-pathogenesis, diagnosis, prognosis and antiviral therapy. AIDS Rev.

[65] Swaminathan G, Navas-Martin S, Martin-Garcia J (2014). MicroRNAs and HIV-1 infection: antiviral activities and beyond. J Mol Biol.

[66] Cox JE, Sullivan CS (2014). Balance and stealth: the role of noncoding RNAs in the regulation of virus gene expression. Ann Rev Virol.

[67] Saayman S, Ackley A, Turner AM, Famiglietti M, Bosque A, Clemson M (2014). An HIV-encoded antisense long noncoding RNA epigenetically regulates viral transcription. Mol Ther.

[68] Chang H, Lim J, Ha M, Kim VN (2014). TAIL-seq: genome-wide determination of poly(A) tail length and 3′ end modifications. Mol Cell.

[69] Weake VM, Workman JL (2010). Inducible gene expression: diverse regulatory mechanisms. Nat Rev Genet.

[70] Voss TC, Hager GL (2014). Dynamic regulation of transcriptional states by chromatin and transcription factors. Nat Rev Genet.

[71] Ocwieja KE, Brady TL, Ronen K, Huegel A, Roth SL, Schaller T (2011). HIV integration targeting: a pathway involving Transportin-3 and the nuclear pore protein RanBP2. PLoS Pathog.

[72] Imbeault M, Giguere K, Ouellet M, Tremblay MJ (2012). Exon level transcriptomic profiling of HIV-1-infected CD4(+) T cells reveals virus-induced genes and host environment favorable for viral replication. PLoS Pathog.

[73] Wan Y, Qu K, Zhang QC, Flynn RA, Manor O, Ouyang Z (2014). Landscape and variation of RNA secondary structure across the human transcriptome. Nature.

[74] Donahue DA, Wainberg MA (2013). Cellular and molecular mechanisms involved in the establishment of HIV-1 latency. Retrovirology.

[75] Lusic M, Giacca M (2014). Regulation of HIV-1 latency by chromatin structure and nuclear architecture. J Mol Biol.

[76] Park J, Lim CH, Ham S, Kim SS, Choi BS, Roh TY (2014). Genome-wide analysis of histone modifications in latently HIV-1 infected T cells. AIDS.

[77] Jadlowsky JK, Wong JY, Graham AC, Dobrowolski C, Devor RL, Adams MD (2014). Negative elongation factor is required for the maintenance of proviral latency but does not induce promoter-proximal pausing of RNA polymerase II on the HIV long terminal repeat. Mol Cell Biol.

[78] Evans VA, Kumar N, Filali A, Procopio FA, Yegorov O, Goulet JP (2013). Myeloid dendritic cells induce HIV-1 latency in non-proliferating CD4+ T cells. PLoS Pathog.

[79] Kapranov P, Cheng J, Dike S, Nix DA, Duttagupta R, Willingham AT (2007). RNA maps reveal new RNA classes and a possible function for pervasive transcription. Science.

[80] Yang L, Duff MO, Graveley BR, Carmichael GG, Chen LL (2011). Genomewide characterization of non-polyadenylated RNAs. Genome Biol.

[81] Marzluff WF, Wagner EJ, Duronio RJ (2008). Metabolism and regulation of canonical histone mRNAs: life without a poly(A) tail. Nat Rev Genet.

[82] Lassen KG, Ramyar KX, Bailey JR, Zhou Y, Siliciano RF (2006). Nuclear retention of multiply spliced HIV-1 RNA in resting CD4+ T cells. PLoS Pathog.

[83] Kim MS, Pinto SM, Getnet D, Nirujogi RS, Manda SS, Chaerkady R (2014). A draft map of the human proteome. Nature.

[84] Zhang L, Zhang X, Ma Q, Zhou H (2010). Host proteome research in HIV infection. Genom, Proteome Bioinforma.

[85] Zhu J, Davoli T, Perriera JM, Chin CR, Gaiha GD, John SP (2014). Comprehensive identification of host modulators of HIV-1 replication using multiple orthologous RNAi reagents. Cell Rep.

[86] Malim MH, Bieniasz PD (2012). HIV restriction factors and mechanisms of evasion. Cold Spring Harb Perspect Med.

[87] DeBoer J, Jagadish T, Haverland NA, Madson CJ, Ciborowski P, Belshan M. Alterations in the nuclear proteome of HIV-1 infected T-cells. Virology. 2014;468-470:409–20. 10.1016/j.virol.2014.08.029 PMC4253593 25240327

[88] Haverland NA, Fox HS, Ciborowski P (2014). Quantitative proteomics by SWATH-MS reveals altered expression of nucleic acid binding and regulatory proteins in HIV-1-infected macrophages. J Proteome Res.

[89] Greco TM, Diner BA, Cristea IM (2014). The impact of mass spectrometry-based proteomics on fundamental discoveries in virology. Ann Rev Virol.

[90] Weekes MP, Tomasec P, Huttlin EL, Fielding CA, Nusinow D, Stanton RJ (2014). Quantitative temporal viromics: an approach to investigate host-pathogen interaction. Cell.

[91] Altelaar AF, Munoz J, Heck AJ (2013). Next-generation proteomics: towards an integrative view of proteome dynamics. Nat Rev Genet.

[92] Jager S, Cimermancic P, Gulbahce N, Johnson JR, McGovern KE, Clarke SC (2012). Global landscape of HIV-human protein complexes. Nature.

[93] Navare AT, Sova P, Purdy DE, Weiss JM, Wolf-Yadlin A, Korth MJ (2012). Quantitative proteomic analysis of HIV-1 infected CD4+ T cells reveals an early host response in important biological pathways: protein synthesis, cell proliferation, and T-cell activation. Virology.

[94.•] Wojcechowskyj JA, Didigu CA, Lee JY, Parrish NF, Sinha R, Hahn BH (2013). Quantitative phosphoproteomics reveals extensive cellular reprogramming during HIV-1 entry. Cell Host Microbe.

[95] Pan X, Baldauf HM, Keppler OT, Fackler OT (2013). Restrictions to HIV-1 replication in resting CD4+ T lymphocytes. Cell Res.

[96] Zack JA, Kim SG, Vatakis DN (2013). HIV restriction in quiescent CD4(+) T cells. Retrovirology.

[97] Berro R, de la Fuente C, Klase Z, Kehn K, Parvin L, Pumfery A (2007). Identifying the membrane proteome of HIV-1 latently infected cells. J Biol Chem.

[98] Yang W, Zhou J-Y, Chen L, Ao M, Sun S, Aiyetan P (2014). Glycoproteomic analysis identifies human glycoproteins secreted from HIV latently infected T cells and reveals their presence in HIV+ plasma. Clin Proteome.

[99] Britton LM, Sova P, Belisle S, Liu S, Chan EY, Katze MG (2014). A proteomic glimpse into the initial global epigenetic changes during HIV infection. Proteomics.

[100] Bartha I, McLaren PJ, Ciuffi A, Fellay J, Telenti A (2014). GuavaH: a compendium of host genomic data in HIV biology and disease. Retrovirology.

